# Posterior Interosseous Fascicular Constriction Within the Radial Nerve in a Diabetic Patient With Bilateral Neuralgic Amyotrophy: A Case Report

**DOI:** 10.3389/fneur.2021.701571

**Published:** 2021-09-08

**Authors:** Woojun Kim, Soo Hwan Kang, Jae Young An

**Affiliations:** ^1^Department of Neurology, Seoul St. Mary's Hospital, The Catholic University of Korea, Seoul, South Korea; ^2^Department of Orthopedic Surgery, College of Medicine, St. Vincent's Hospital, The Catholic University of Korea, Seoul, South Korea; ^3^Department of Neurology, College of Medicine, St. Vincent's Hospital, The Catholic University of Korea, Seoul, South Korea

**Keywords:** ultrasound, fascicular constriction, posterior interosseous nerve, radial nerve, neuralgic amyotrophy

## Abstract

**Background:** Neuralgic amyotrophy (NA) is an acute, monophasic, painful inflammatory dysimmune focal, or multifocal mononeuropathy. The lesion in NA is not always restricted to the brachial plexus but also involves individual nerves or branches. The prognosis of NA is less favorable than previously assumed, but the reasons for poor recovery remain unknown. Nerve constriction may be one of the causes of poor prognosis in NA.

**Case Presentation:** Herein, we described a 54-year-old male with a history of type 2 diabetes in whom bilateral neuralgic amyotrophy developed with constriction of the posterior interosseous fascicle within the radial nerve. The patient experienced sudden-onset severe pain in both shoulders followed, 2 days later, by weakness in bilateral shoulders and the left forearm extensors over the subsequent month. The left forearm extensors were more severely affected than both shoulder girdle muscles. He noted a 7-kg weight loss for 1 month before pain onset. After diagnosing diabetic NA based on the clinical symptoms, imaging, and electrophysiological studies, treatment with systemic steroids improved pain and weakness in both shoulder muscles. Weakness in the left forearm extensors persisted after 1 month of steroid treatment. Follow-up ultrasound revealed constriction of the posterior interosseous fascicle within the main trunk of the left radial nerve at the elbow. Surgical exploration at 6 months after onset identified fascicle constriction, for which neurolysis was performed. Weakness in the extensors of the wrist and fingers did not improve during the 16-month follow-up.

**Conclusion:** A single constriction of the fascicle within a peripheral nerve may often be under-recognized if NA presents with variable degrees of weakness in bilateral upper limbs. Furthermore, fascicular constriction without edema of the parent nerve may be easily missed on the initial ultrasound. A lack of early recognition of nerve constriction and delay in surgical intervention can result in unfavorable outcomes. The physician should consider the possibility of the fascicular constriction when evaluating patients suspected of brachial NA with significant weakness in the distal upper limb compared to the proximal weakness or weakness of the distal upper limb that does not improve over time.

## Introduction

Idiopathic neuralgic amyotrophy (NA), also known as Parsonage–Turner syndrome or brachial plexus neuritis, is characterized by extreme pain at symptom onset, rapid multifocal paresis, atrophy of the upper extremity muscles, and a slow recovery requiring months to years (1). Although the classical presentation is found in about two-thirds of patients, NA can also manifest with the involvement of a single peripheral nerve or various combinations of peripheral nerves of the brachial plexus (2). This clinical variation that also involves the nerve of non-brachial plexus origin, such as accessory nerve, has led to the concept of NA that encompasses all these acute-onset, painful focal, or multifocal neuropathies with a monophasic course (1–3). The prognosis of NA was traditionally thought to be favorable, with pain subsided within a few weeks and weakness improved over months to years. However, based on a large series of patients with NA, overall recovery was less favorable than previously assumed (4). The exact cause of the unfavorable outcome is unknown. However, previous studies reported surgical findings of nerve constriction in patients with typical symptoms of NA and no spontaneous recovery (5, 6).

Here, we report a case of a diabetic male with bilateral brachial NA showing posterior interosseous fascicular constriction within the radial nerve on ultrasound, and which was confirmed by surgical exploration.

## Case Presentation

**Figure 1:**
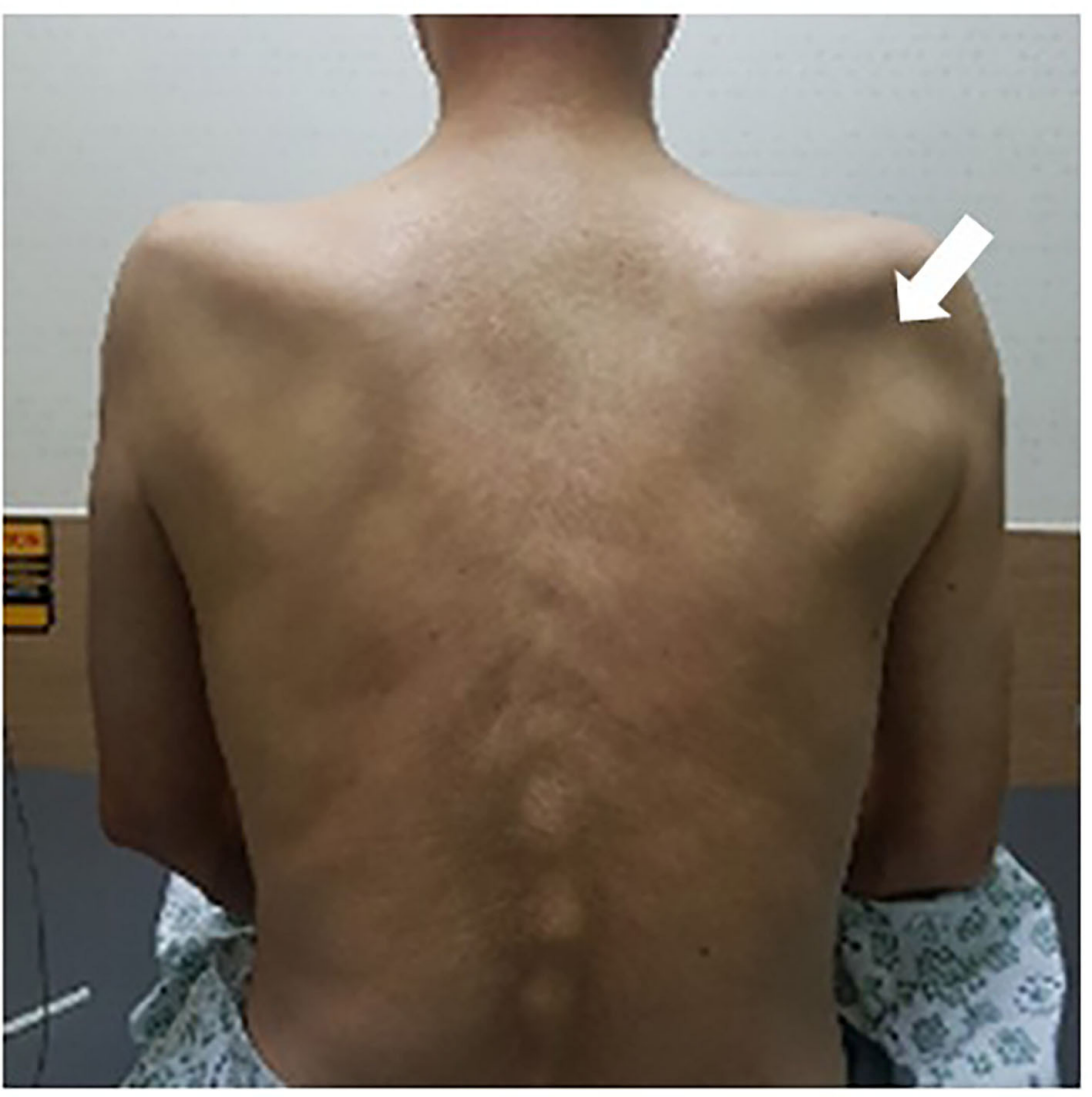
Muscle atrophy of bilateral supraspinatus and infraspinatus. The bilateral infraspinatus fossae are dented, with greater severity on the right side (arrow).

**Figure 2:**
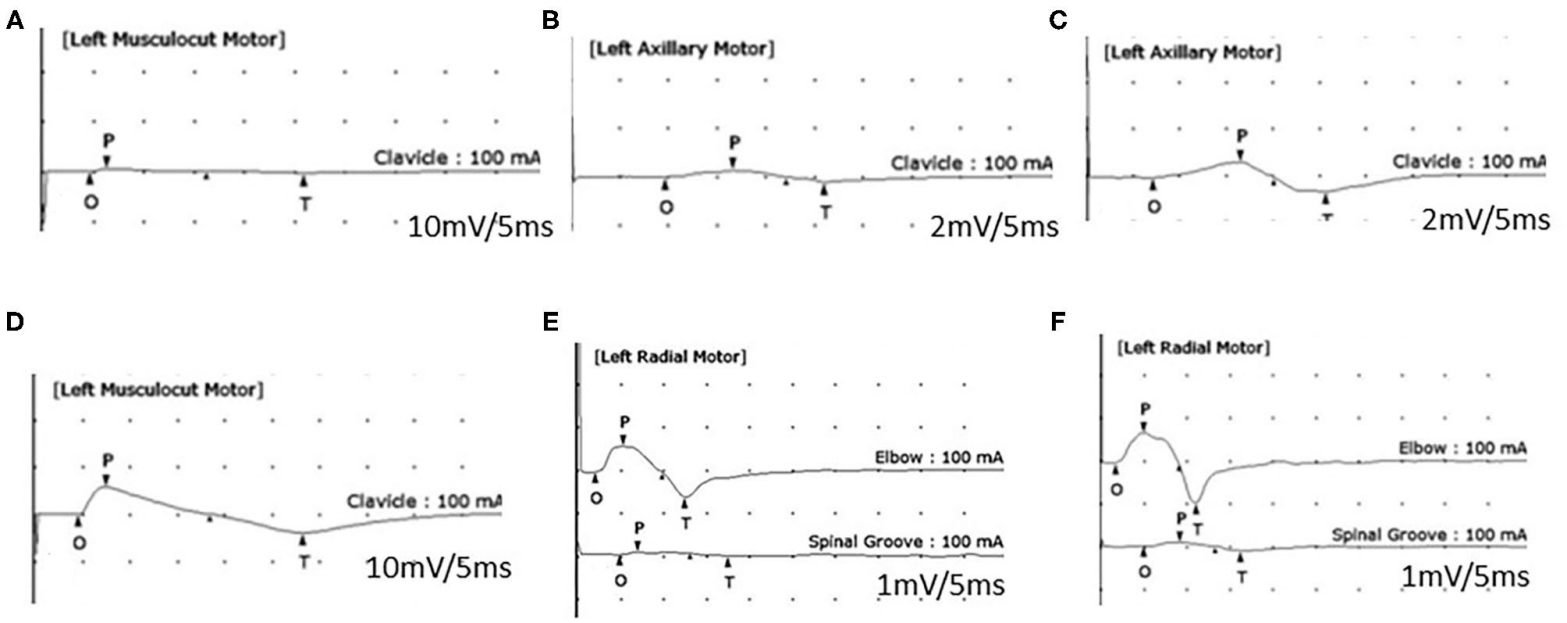
Motor nerve conduction study (NCS). An initial NCS **(A,B)** shows a reduced amplitude of compound muscle action potentials (CMAPs) of the left musculocutaneous nerve (**A**: 1.1 mV, recording of the biceps brachii muscle) and left axillary nerve (**B**: 0.5 mV, recording of the deltoid muscle). A 1-month follow-up NCS **(C–E)** shows the improved amplitude of CMAPs of the left axillary nerve (**C**: 10.2 mV), the left musculocutaneous nerve (**D**: 13 mV), and a conduction block of the left radial nerve (recording of the extensor indicis muscle) between the elbow and spiral groove **(E)**. A 5-month follow-up NCS **(F)** reveals a persistent conduction block of the left radial nerve.

**Figure 3:**
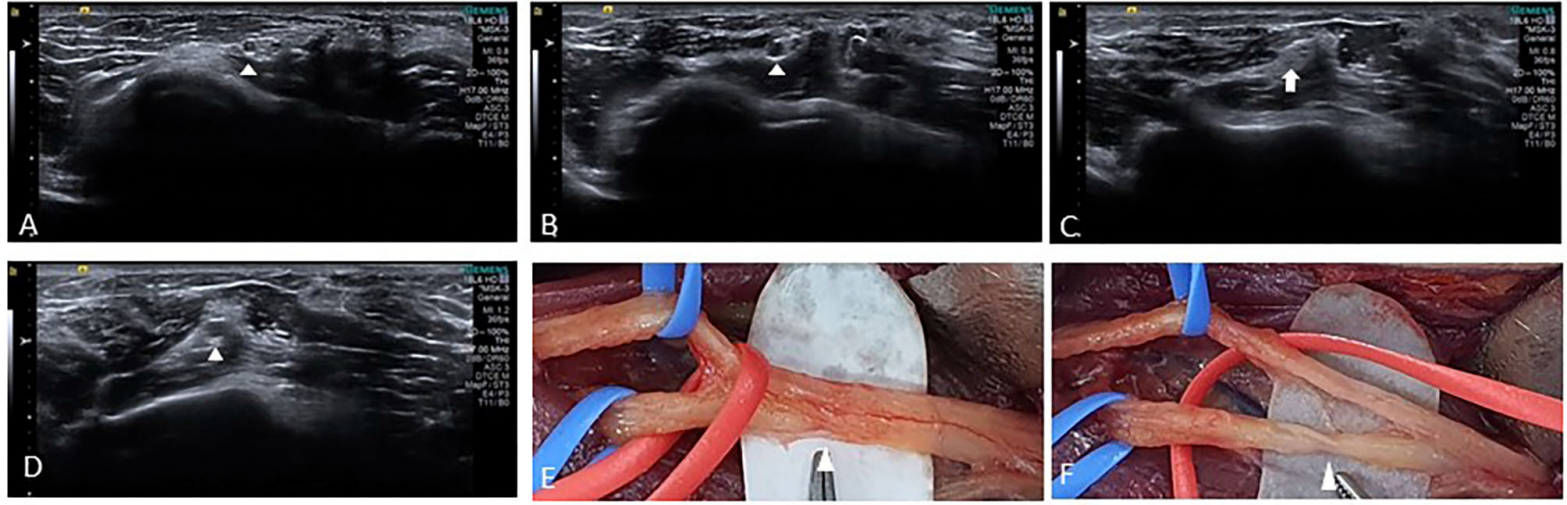
Ultrasound images from cine clips of the left radial nerve in the short axis show a normal posterior interosseous fascicle (arrowhead) within the radial nerve **(A)**, swelling (arrowhead) is noted as the nerve is traced proximally **(B)**, followed by fascicular constriction (arrow) **(C)**, and a swollen fascicle (arrowhead) proximal to the constriction **(D)**. Intraoperative photograph shows partial constriction **(E)** of radial nerve before branching posterior interosseous and superficial radial sensory nerves proximal to the lateral epicondyle. After neurolysis, constriction **(F)** of the posterior interosseous fascicle was discovered.

A 54-year-old man was admitted with a 2-month history of pain and weakness in both shoulders. The patient was an office worker with no past medical history except for well-controlled hypertension and diabetes for 10 years. Two months prior, he complained of sudden onset of pain in both shoulders and developed the weakness of both shoulder girdles 2 days later. The weakness in the left shoulder girdle progressed, and weakness in the left wrist and finger extensors newly developed over the next 4 weeks. There were no preceding trauma, immunization, or fever, but he reported weight loss (7 kg) over the 1 month before pain onset. The patient had never regularly exercised other than walking for glycemic control. The symptoms did not change for the next 1 month before admitting to our hospital. A neurological examination showed paresis of shoulder abduction [right: medical research council grade (MRC) 4/5, left: MRC 3/5], elbow flexion (right: MRC 4/5, left: MRC 3/5), elbow extension (right: MRC 4/5, left: MRC 3/5), wrist flexion (right: MRC 4/5, left: MRC 4/5), wrist extension (right: MRC 4/5, left: MRC 2/5), and extension of fingers (right: MRC 4/5, left: MRC 2/5). The muscle power of bilateral finger flexion and lower limbs was normal. A hypesthesia area was identified in the lateral sides of bilateral shoulders and arms, and deep tendon reflexes were reduced in the upper limbs. Atrophy of both shoulder girdles was observed (Figure 1). A complete blood cell count and routine biochemical analysis were normal except for fasting glucose (151 mg/dl) and HbA1c (7.3%). Other serological tests for human immunodeficiency virus, hepatitis B, C, and E, syphilis, and autoimmune diseases including angiotensin-converting enzyme, anti-nuclear antibody, double-stranded DNA, anti-Ro, anti-La, and anti-neutrophil cytoplasmic antibody were negative. An initial nerve conduction study (NCS), performed 2 months after symptom onset, showed a decrease of compound muscle action potential amplitude in the left musculocutaneous nerve (Figure 2A, recording of the biceps brachii muscle) and axillary nerve (Figure 2B, recording of the deltoid muscle) and a significant asymmetry of sensory nerve action potentials of the left lateral antebrachial cutaneous nerve (2.0 μV) compared to the right side (5.0 μV). NCS findings of the bilateral median, ulnar, peroneal, tibial, and sural nerves were normal. Needle electromyography (EMG) showed various degrees of denervation potentials in bilateral infraspinatus, supraspinatus, left deltoid, left triceps, left extensor digitorum, and left flexor carpi radialis muscles. There was reduced recruitment of motor unit in bilateral serratus anterior and right deltoid muscles. The results of initial electrophysiological studies suggested the involvement of bilateral upper and middle trunks (C5–C7) of the brachial plexus, particularly a more severe involvement of the left brachial plexus. Ultrasound with a 18–6-MHz linear transducer was performed on the same day as NCS/EMG and showed no swelling in the brachial plexus, median, radial, ulnar nerves. Magnetic resonance imaging of the brachial plexus was carried out 3 days after NCS/EMG, showing mild swelling without enhancement at the cord level of the left brachial plexus. Based on the clinical history of the acute intense pain of shoulders and weakness of upper limbs with preceding weight loss, considering the involvement of bilateral upper and middle branches of brachial plexus in the electrophysiological studies and having excluded other causes by imaging and serological studies, a diagnosis of diabetic NA was made. Oral steroid (1 mg/kg) was administered 9 weeks after symptom onset and tapered over 4 weeks. Bilateral shoulder pain and weakness, except for weakness of the left wrist and finger extensors, improved. In the follow-up study of the left axillary (Figure 2C) and musculocutaneous (Figure 2D) nerves 4 weeks after the initial study, the amplitude of compound muscle action potential was improved. However, on the conduction study of the left radial nerve that was not included in the initial study, we detected a conduction block between the spiral groove and the elbow (Figure 2E, recording of the extensor indicis muscle). The nerve action potentials of bilateral superficial radial sensory nerves were symmetric. A 4-week follow-up ultrasound revealed constriction of the left posterior interosseous fascicle within the left radial nerve at 1.5 cm proximal to the lateral epicondyle (Figures 3A–D). Surgical exploration for nerve constriction was considered but refused by the patient. Therefore, low-dose oral steroids and a rehabilitation program were continued for another 2 months. A 5-month follow-up NCS revealed a persistent conduction block of the left radial nerve (Figure 2F). The weakness of the left wrist and finger extensors was unchanged (MRC 2/5), but other remaining upper limb muscles were normal, except for the left shoulder abduction (MRC 4/5), left elbow flexion (MRC 4/5), and extension (MRC 4/5). Surgical exploration was performed 6 months after symptom onset and confirmed fascicular constriction at the area identified by ultrasound, for which interfascicular neurolysis was performed (Figures 3E,F). During the 16-month follow-up, the weakness in the wrist and finger extensors did not improve (MRC 2/5). We considered a tendon transfer to improve hand function a year after neurolysis, but the patient did not want secondary surgery.

## Discussion

Brachial NA remains unfamiliar to many physicians who often diagnose such patients with glenohumeral bursitis or cervical radiculopathy. Such a delay in diagnosis of NA can lead to suboptimal treatment in the acute stage (2, 4). The minimum incidence of NA is one to three cases per 100,000 individuals in the general population (7, 8); however, under-recognition and misdiagnosis are frequent, and the incidence recently has been estimated to be 1 per 1,000 per year (9). The precise pathophysiological mechanism remains unclear but is thought to result from an underlying genetic predisposition, a susceptibility to mechanical injury, and immune or autoimmune triggers for the attacks (2). Patients with diabetes mellitus sometimes present with acute or subacute, progressive, asymmetrical pain, and weakness of the proximal lower limb muscles, known as diabetic amyotrophy, Bruns-Garland syndrome, or diabetic lumbosacral radiculoplexus neuropathy (RPN) (10). Diabetic RPN can affect the upper limb, which shares many clinical features with NA (11). Massie et al. reported that diabetic cervical RPN had autonomic features, more frequent weight loss, co-occurring thoracic and lumbosacral RPN, more involvement outside of the brachial plexus, and more involvement of the lower trunk of the brachial plexus (11). However, other researchers suggested that NA in diabetic patients is merely a chance occurrence (2, 4). Recent studies suggested that NA and diabetic RPN are variants of non-systemic vasculitic neuropathies, but whether diabetic NA should be separated from idiopathic NA is a matter of ongoing debate (12, 13).

In several recent studies, persistent pain and paresis were experienced by up to two-thirds of patients with idiopathic NA (1, 2). Since first reported by Abe et al. (14) nerve constriction has been established as an unexplained surgical finding in various nerves of the upper extremity (15). Pan et al. first reported a surgical finding of hourglass-like constriction of the upper-extremity nerves in patients with typical symptoms of NA and no spontaneous recovery (6). Surgical treatment resulted in generally good recovery, and this nerve constriction or nerve torsion may be a clue for poor prognosis in some patients with NA (3, 15). The pathogenesis of nerve constriction and torsion is unclear but is considered due to local inflammation, which can cause intrafascicular edema, adhesion, and local fixation of fascicles, resulting in the thinning and constriction of nerve and making the nerve susceptible to torsion (5, 16, 17).

NA has been considered a predominantly clinical diagnosis, based on the characteristic history and physical findings, (1, 4) and laboratory tests are of little diagnostic value (2). Many clinicians are generally considering electrophysiological studies as the first test to diagnose brachial NA. However, the sensitivity of NCS for this disorder is very low, and EMG commonly produces negative results due to sampling error (18). With the introduction of improved imaging methods such as magnetic resonance imaging and high-resolution ultrasound in the diagnostic workup of NA, distinct structural nerve pathologies have been identified as pathognomonic in NA patients (5, 6, 15, 19, 20). Arányi et al. identified abnormal ultrasound findings in 74% of patients with NA (3). In their report, four types of abnormalities were classified as swelling without constriction, swelling with incomplete constriction, swelling with complete constriction, and fascicular entwinement. Nerve swelling is recognized easily on ultrasound, irrespective of constriction, and so is rotational nerve torsion. However, fascicular constriction within the parent nerve can be missed in the ultrasound without high-index suspicion.

Although it is still controversial when surgical treatment should be performed, surgical treatment should be considered for patients who did not show spontaneous recovery by 3 months after onset (5, 12, 21–23). Furthermore, if severe constrictions with nerve torsion and fascicular entwinement are identified through ultrasound before the 3-month interval, early intervention may be justified as spontaneous recovery is not to be expected in these cases (3). The choice of optimal surgical treatment for the patient with nerve constriction depends on the age, delay repair interval, and severity of constriction (23, 24). Surgical treatments include interfascicular neurolysis, tendon transfer, and neurorrhaphy or autografting. Intrafascicular neurolysis is suggested for mild to moderate constriction and nerve reconstruction for severe constriction (12, 23). Previous studies have noted that younger patients had a higher chance for good recovery, while patients aged 50 or older more frequently showed unfavorable results (12, 23, 24). Moreover, Ochi et al. suggested interfascicular neurolysis together with tendon transfer for patients over 50 years old with no sign of recovery or the younger group with a delay repair interval of more than a year (24).

The development of nerve constriction irrespective of torsion in brachial NA might be a sign of poor prognosis (15). We did not include radial nerve in the initial NCS, as we thought that asymmetric brachial plexus lesions caused prominent weakness of the left forearm extensors at the initial examination, and we were belatedly aware of the left radial nerve lesion due to persistent weakness of the left forearm extensors. Furthermore, the initial ultrasound was unable to identify fascicular constriction because fascicular constriction occurred in short segments without edema of the main trunk of the radial nerve. Even if we cannot determine which of the delay of the surgery, old age of the patient, or method of surgical treatment affected the poor prognosis through our single case, early detection of fascicular constriction is the essential step in optimizing the management of NA patients with nerve constriction. Therefore, a careful ultrasound examination needs to be performed in the initial evaluation for NA, and follow-up ultrasound for fascicular constriction should be considered if prominent distal weakness is present compared with proximal weakness or if there is the distal weakness that does not improve after treatment.

## Data Availability Statement

The original contributions generated for the study are included in the article/Supplementary Material, further inquiries can be directed to the corresponding author/s.

## Ethics Statement

The studies involving human participants were reviewed and approved by The catholic university of Korea, St. Vincent's Hospital institutional Review Board. The patients/participants provided their written informed consent to participate in this study. Written informed consent was obtained from the individual(s) for the publication of any potentially identifiable images or data included in this article.

## Author Contributions

WK, SK, and JA take responsibility of the study concept, and design, integrity of the data, drafting of the manuscript, and study supervision. SK and JA collected and analyzed patient information. All the authors read and approved the manuscript.

## Conflict of Interest

The authors declare that the research was conducted in the absence of any commercial or financial relationships that could be construed as a potential conflict of interest.

## Publisher's Note

All claims expressed in this article are solely those of the authors and do not necessarily represent those of their affiliated organizations, or those of the publisher, the editors and the reviewers. Any product that may be evaluated in this article, or claim that may be made by its manufacturer, is not guaranteed or endorsed by the publisher.
